# Beyond This Book

**DOI:** 10.1007/978-3-030-56282-3_8

**Published:** 2020-08-28

**Authors:** Nicola Morelli, Amalia de Götzen, Luca Simeone

**Affiliations:** Aalborg University, Copenhagen, Denmark

## Abstract

Some concluding notes originate from the view proposed by the previous chapters. This particular view is like a picture that captures a portion of a landscape in a specific frame, time and logical context. This chapter clarifies this frame and suggests ways to extend this view beyond the logical, professional and time limitations that a book could possibly present.

This book offers an overview of the capabilities that a service designer should acquire to be able to qualify their contribution to society. Many other possible topics have been left out of this book because, although very relevant, they are not specific to the profession of a service designer. Furthermore, the reader should take into account that a book is a picture of a context in time, where the context is not static and is extended much beyond the frame and the time of the picture. Certain considerations, which are outlined in the next section, position the book in respect to the professional, logical and historical context in which a service designer would operate.

## Beyond service design competence

This book is grounded on the belief that designers’ capabilities need to be complemented with other professional capabilities that are essential for consolidating innovation processes. The collaboration between design and professionals from other disciplines, such as technical experts, anthropologists, sociologists or management experts, has been implicitly assumed in this book. Another relevant aspect that has only been marginally touched upon in this book is the evaluation of the impact of design action. The many toolkits and handbooks on how to design services does not correspond with any quality research on how such tools are used and the extent to which the use of such tools has been effective. However, this is hardly surprising, given that service design capabilities are generally not measured according to any quantitative parameters. Such a quantitative measurement could clearly validate the contribution of designers in innovation processes according to a logic that could help convince investors or the financial departments of public institutions. The parameters of such a quantitative measurement have never been defined and probably cannot be defined in absolute terms, although studies have been conducted that focus on the criteria and strategies for an evaluation of services from mixed quantitative and qualitative perspectives (Foglieni et al. 2013, 2018).

## Beyond the frame

As mentioned in the first sections, the aim of this book was not to create a new design manual for our students, as many and better manuals that offer detailed descriptions and examples of how service design tools are used are already available. Instead, the aim was to provide a navigation tool to read service design action in relation to the capabilities that are now required of service designers.

The authors discovered that although several tools are available for operating in the growing area of service design, some concepts and perspectives of this discipline, and on innovation in general, have not been properly integrated into a methodological approach. This encouraged the authors to attempt to see how the role and capabilities of service designers can be described in the light of two fundamental logical changes:

- The first change concerns the way to view value production in services, as introduced and explored by the service dominant logic. Such changes in fact shift the main role in value production from service providers (and a designer supporting them) to service beneficiaries. When looking at designers’ capabilities and roles, this change prompts the question—*what is the role of designers, and what capabilities should they have in the service dominant logic?*
- A logical change deriving from a multi-level perspective that observes innovation paths at different scales. This perspective suggests the question—*how are service design capabilities used at different levels of innovation?* In this respect, this book proposes a navigation map that would help them understand which tool to use and how to use it according to the scale of change they are operating to.

This is the point of this book. And the authors believe this approach will also help support designers when navigating the complexity of their profession in light of the changes to come.

## Beyond the (present) time

This book is being written in an historical moment characterised by a complex and possibly revolutionary change that will leave a deep footprint on the way our society is organised.

In ‘normal’ times, the operative domain of design would be mostly limited to changes at the levels of ‘service as interaction’ and of ‘service as infrastructure’. Most of the academic contributions on innovation processes are based on the assumption that the overall cultural, social, economic and political landscape in which our living systems are organised are changing at a very slow pace and according to evolutionary logics in which human beings, and therefore, designers, have very little control.

Many authoritative sources in innovation literature, however (Schumpeter 1943; Dosi 1982; Kuhn 1962) point out that the history of innovation is not a constant progress but rather proceeds by alternating long periods of slow development and short periods of revolutionary changes. The hypothesis that the COVID-19 crisis, and its planetary character, may be one of those revolutionary changes would need an in-depth analysis, which is not in the scope of this book. However, some hypotheses can be made on the nature of the coming change that have a much more operative character.

The crisis is prospecting a radical change in relationships between people, the organisation of services, and future technological, economic and environmental policies, and perhaps, in the long term, this change will also influence political systems. This change cannot be predicted, but we need to prepare our eyeglasses to properly observe and interpret them. Indeed, multifocal glasses will be needed for a close look at the changes in the way we live and also for a distanced look on the way we interpret and reorganise our world.

As for any pair of multifocal glasses, the structure of the lens makes it possible to focus on different distances and scales. In line with this, the logical structure of this book proposes a similar observation tool that allows for an interpretation and mapping of different levels of reality and suggests different capabilities and tools that make it possible to navigate in such a reality.
